# One-and-done middle meningeal artery embolization with next-generation optiblock coils: streamlining workflow for chronic subdural hematoma

**DOI:** 10.1007/s00234-025-03874-w

**Published:** 2026-01-05

**Authors:** Sarah Hamimi, Jaeha Kim, Aaron Anandarajah, Nathan Yu, Suraj Dumasia, Julia Ognibene, Arti Singh, Mikaeel Habib, Linda Bagley, Omar A. Choudhri

**Affiliations:** 1 University of Pennsylvania, Philadelphia, USA; 2 University of Rochester, Rochester, USA; 3 Weill Cornell Medicine, New York City, USA

**Keywords:** Subdural hematoma, MMA embolization, Coils, Neurointerventional radiology

## Abstract

**Purpose:**

Middle meningeal artery (MMA) embolization is an adjunct or alternative to surgery for chronic subdural hematoma (cSDH), but data on coil-forward strategies remain limited. The purpose of this study is to evaluate safety and outcomes after MMA embolization using Optiblock coils engineered for efficient mechanical vessel occlusion.

**Methods:**

Clinical data was extracted for a retrospective cohort of adults treated from February 2023 to December 2024 with ≥ 1 Optiblock coil; adjunctive embolic agent allowed. Symptoms, modified Rankin Scale (mRS), maximal SDH thickness, and midline shift were assessed at serial timepoints. McNemar’s test and the Friedman test were used for longitudinal comparisons, with α = 0.05.

**Results:**

Thirty-one patients underwent embolization (mean age 74.0; 84% male). Procedures used a mean of 1.28 coils with an average total coil length of 27.2 cm. The most common Optiblock coil used was 3.5 mm x 20 cm. Embospheres (MERIT medical) were used as an adjunct in 87% of cases. Mean SDH thickness decreased from 14.8 mm pre-operatively to 8.9, 4.4, and 2.1 mm at ~ 1, 3, and 6 months post-operation (*p* < 0.05), corresponding to 40%, 71%, and 86% reductions; midline shift decreased concordantly (*p* < 0.05). mRS improved from 2.2 at baseline to 0.6 and 0.1 at first and last follow-up, respectively (*p* < 0.001). No periprocedural complications were noted. Interval surgical drainage was performed in 2 patients (6.5%) after initial isolated MMA embolization. Two delayed deaths occurred, both from known cardiovascular conditions unrelated to embolization.

**Conclusion:**

Optiblock-based MMA embolization was associated with low complication and reintervention rates and substantial clinical, functional, and radiographic improvement. These coils offer an efficient alternative and/or adjunct to liquid/particle embolization, particularly in cases where particle or liquid embolization is contraindicated or technically unfeasible.

**Supplementary Information:**

The online version contains supplementary material available at 10.1007/s00234-025-03874-w.

## Introduction

Chronic subdural hematoma (cSDH) is a widely prevalent neurosurgical condition, particularly among older adults [1]. Incidence of cSDH is expected to rise with an aging population and increasing use of anticoagulation and antiplatelet agents. The standard of care for symptomatic cSDH has traditionally been surgical evacuation alone. While generally effective, particularly in the short term, surgical hematoma evacuation is associated with recurrence rates ranging from 10% to 20% and often requires repeat interventions, especially in patients with ongoing risk factors such as brain atrophy or coagulopathies [2–4].

Recent insights into the pathophysiology of cSDH have shifted the therapeutic focus from surgical hematoma evacuation alone to addressing the vascularized neomembranes that surround the hematoma. cSDH is increasingly understood as a dynamic inflammatory process initiated by fibrinolysis of an initial hemorrhage, which triggers recruitment of inflammatory cells, dural thickening, and aberrant repair. This maladaptive response leads to angiogenesis and the formation of immature, fragile capillaries prone to leakage. The persistent exudation from these neovessels—largely supplied by branches of the middle meningeal artery (MMA)—exceeds the rate of resorption, contributing to hematoma expansion and chronicity [5–7]. Accordingly, endovascular embolization of the MMA has emerged as a minimally invasive technique designed to occlude the blood supply to these vascular membranes, thereby promoting hematoma resorption and potentially reducing recurrence. There is now level 1 clinical evidence demonstrating the efficacy of MMA embolization for cSDH as a surgical adjunct and as a standalone therapy [8–11]. While several studies have demonstrated reduced recurrence rates and improved outcomes with MMA embolization, other studies have questioned its added benefit over standard surgical management alone [12–16].

Various embolic materials have been employed for MMA embolization, including polyvinyl alcohol (PVA) particles, liquid embolics such as Onyx, and metallic coils [17]. Particle and liquid embolic agents are favored for their ability to penetrate distal branches of the MMA and occlude the fragile neovasculature within the hematoma membranes. However, their low viscosity increases the risk of reflux into dangerous anastomoses, which are connections between the MMA and adjacent arterial systems such as the ophthalmic, facial, or internal carotid arteries. Reflux into these vessels may result in non-target embolization, leading to serious complications such as vision loss, cranial nerve deficits, or stroke. In contrast, coil-based embolization offers several safety advantages. Coils provide mechanical occlusion with limited distal spread and are deployed under direct fluoroscopic guidance, minimizing the likelihood of reflux. Despite these advantages, traditional coils have been constrained by their short lengths and low packing densities, often necessitating multiple deployments to achieve durable occlusion. Recent studies also suggest that combining coils with particles may enhance efficacy by enabling delivery of a greater volume of embolisate into the MMA [18]. A few studies have investigated the efficacy of coil embolization of the MMA as standalone strategy or as an adjunct with liquid or particulate embolic agents for cSDH, showing promising results [19–21].

**Fig. 1:**
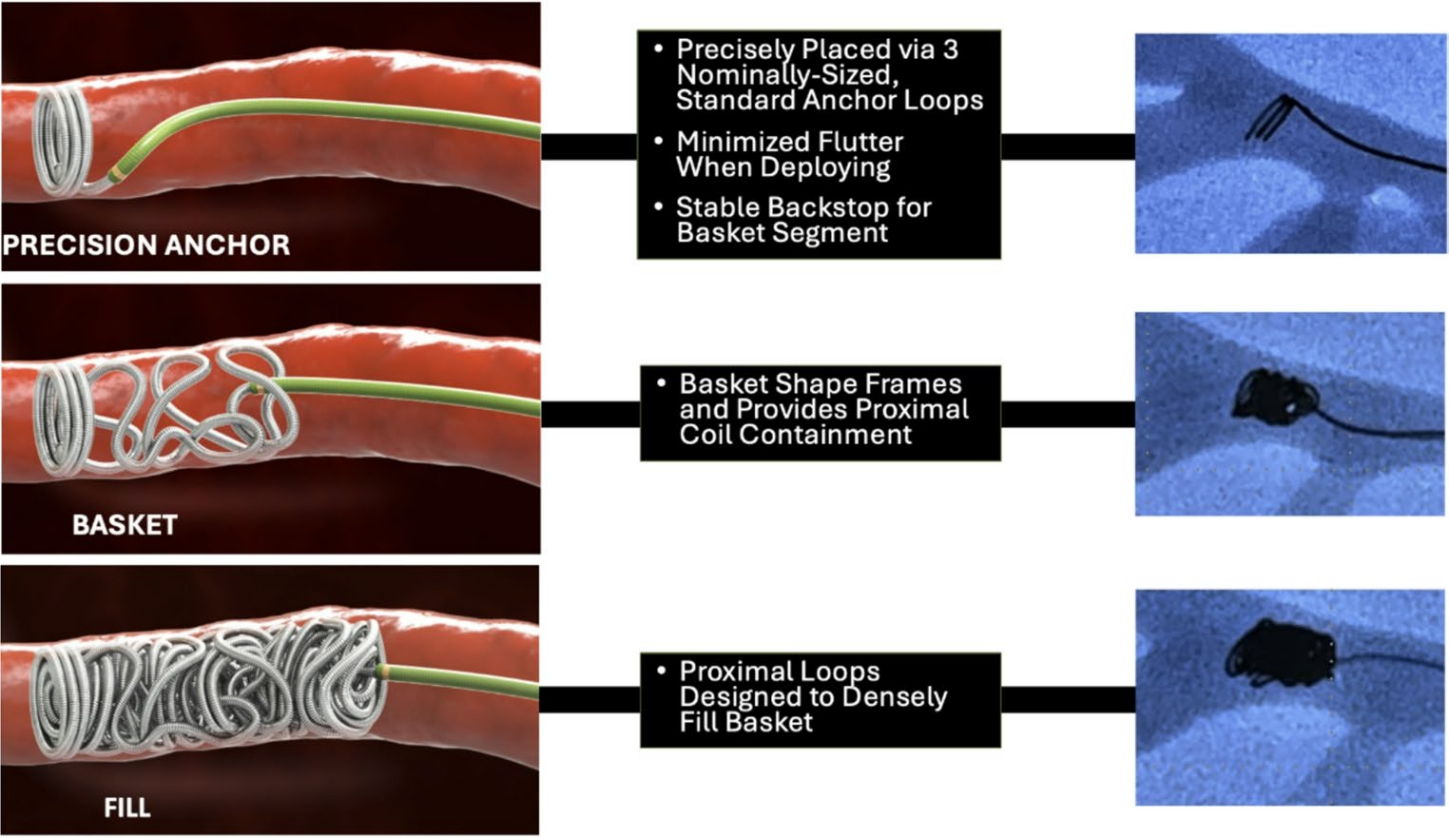
Illustration demonstrating the stepwise deployment of Optiblock coils (Balt USA, Irvine, CA). Figure created by Balt Group USA. Used with permission

The Optiblock coil (Balt USA, Irvine, CA, Fig. 1) is a novel tool designed to address some of these limitations. It features an integrated 3-in-1 structure composed of an anchoring segment, a basket segment, and a thrombogenic filler coil, all welded into a single elongated unit. With lengths up to 65 cm—more than triple that of traditional coils—Optiblock enables high packing density and efficient vessel occlusion with fewer deployments. Furthermore, its design facilitates dense, proximal mechanical embolization while minimizing the risk of distal migration or non-target embolization. To our knowledge, no prior clinical studies have specifically evaluated the safety or efficacy of Optiblock in MMA embolization for subdural hematomas.

In this study, we report the first clinical experience using Optiblock coils for MMA embolization in patients with cSDH and subacute SDH. We describe patient demographics, procedural characteristics, clinical and functional outcomes, and longitudinal radiographic changes. We hypothesize that Optiblock-based embolization is a safe, effective and an efficient tool, offering favorable hematoma resolution and functional recovery with low recurrence and complication rates.

## Methods

We performed a retrospective study of patients who underwent MMA embolization for SDHs between February 2023 and December 2024 by three neuro-interventionalists, including the senior author OC who performed a large majority of the embolization procedures (28 out of 31 cases). The procedures were conducted at two centers: the Hospital of the University of Pennsylvania and Virtua Our Lady of Lourdes Hospital, a strategic partner of University of Pennsylvania Health System. Institutional Review Board approval was obtained prior to data collection, and all patients gave informed consent for their procedures.

Eligible patients were adults diagnosed with SDH on non-contrast computed tomography (CT). Patients were included if they underwent MMA embolization, either as a primary intervention or as an adjunct to surgical evacuation. Inclusion criteria also included at least an attempt by the surgeon to use one or more Optiblock coil during the procedure. No patients were excluded from the above cohort.

Clinical decision-making regarding the indication for MMA embolization was left to the treating neurosurgeon, based on symptom severity, imaging features, and recurrence risk. Some patients underwent embolization following surgical evacuation, while others received embolization as the sole therapy. Procedures were performed predominantly under general anesthesia (> 90%), with a minority completed under conscious sedation. Anesthesia modality was selected at the discretion of the treating clinician based on patient factors, clinical context, and whether concurrent burr-hole drainage for SDH was planned. Patient demographics data, procedural metrics, imaging results, and clinical outcomes were retrieved from the electronic health record and documented. Demographic variables recorded included patient age, sex, comorbidities, antiplatelet/anticoagulant use, and anti-epileptic drug use. Data regarding the following comorbidities was retrieved: hypertension, hyperlipidemia, diabetes mellitus, atrial fibrillation or atrial flutter, thyroid disease, coronary artery disease, congestive heart failure, cancer, chronic kidney disease, stroke, thrombocytopenia, dementia, human immunodeficiency virus (HIV), hepatitis C virus (HCV), deep venous thrombosis (DVT), and prior ventriculoperitoneal shunt. Additionally, data regarding the patients’ history of head trauma and presenting symptoms were collected. These symptoms included: headache, gait instability, altered mental status (AMS), dizziness, seizures, motor weakness, aphasia, and altered sensation. Preoperative CT scans and associated imaging reports were reviewed and data regarding laterality of the hematoma, location of the hematoma, size of the hematoma and the presence and extent of midline shift were recorded. Furthermore, the patient’s preoperative notes were reviewed and a modified Rankin Scale (mRS) score was recorded to quantify the level of the patient’s disability period.

**Fig. 2:**
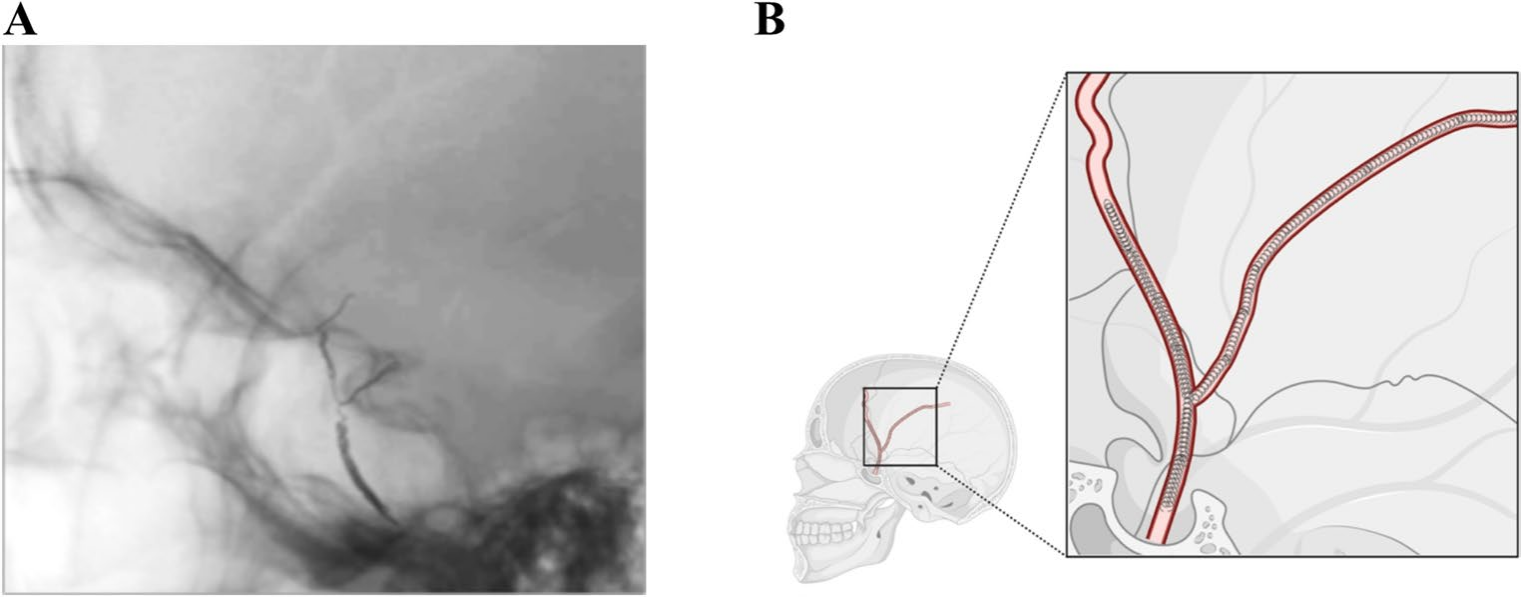
Optiblock coil deployment in the middle meningeal artery (MMA) (**A**) Lateral X-ray showing a single 3 mm × 20 cm Optiblock coil occluding the frontal and parietal branches as well as the common trunk of the MMA. (**B**) Schematic illustration demonstrating Optiblock coil placement within the MMA. Figure created using Biorender.com

All embolization procedures were performed by the senior author using either transradial or transfemoral arterial access. Following selective catheterization of the external carotid artery and superselective angiography of the MMA, embolization was performed using embospheres (100–300 microns) (MERIT Medical, Utah) performed using 0.0165 microcatheter SL-10 (Stryker) distally in the MMA frontal branch in combination of Optiblock coils placed proximally through the same microcatheter after it had been cleared of particles with saline under fluoroscopy (Balt USA, Irvine, CA) (Fig. 2) (Supplementary Fig. 1). Coils were deployed into the frontal and/or parietal branches of the MMA, with the goal of achieving dense mechanical occlusion. If there was concern for dangerous ophthalmic artery anastomosis based on pre-embolization MMA microcatheter injections, embospheres were not employed and Optiblock coils were used in isolation. In a small group of patients, only the common trunk of the MMA was occluded using Optiblock coils in conjunction with other coils or N-butyl cyanoacrylate (nBCA).

Procedural variables, including number of coils used, fluoroscopy time, and radiation skin dose, were recorded for each case. In addition, the size of the coil used, and location and coverage of the Optiblock coil(s) were also recorded.

Patients were evaluated at standard clinical follow-up intervals after embolization, with symptom status and functional performance documented at each visit. Functional outcomes as measured by the mRS scores were extracted from the EMR at the first and most recent follow up visit for each patient if it was available. Symptoms at these follow-up visits—including headache, gait instability, weakness, dizziness, aphasia, and AMS—were also extracted from the EMR.

Non-contrast head CT scans were reviewed preoperatively and at 1-, 3-, and 6-month intervals post-embolization when available. Imaging parameters included maximal subdural hematoma thickness and midline shift, as measured on axial slices. These metrics were extracted from the reports written by an independent neuroradiologist with no association with this study. Recurrence was defined as hematoma re-accumulation associated with worsening clinical symptoms or increased hematoma thickness requiring repeat surgical or endovascular intervention. Complications were defined as any adverse event related to the surgical or interventional procedures.

Descriptive statistics were used to summarize baseline demographic characteristics, comorbidities, and procedural details. Continuous variables were reported as means with standard deviations, and categorical variables as counts and percentages. Comparisons of clinical symptoms across timepoints were made using McNemar’s test for paired categorical data. For several symptom categories, the number of discordant pairs between time points was too small to support a valid McNemar’s test; for these variables, p-values are not reported and are designated as ‘–’ in the tables.

Functional outcomes and radiographic measurements at multiple timepoints were compared using the Friedman rank-sum test and student’s paired t-tests respectively.

Statistical analyses were performed using R (version 4.5.0). A significance level of α = 0.05 was used for all tests.

## Results

A total of 31 patients with known subdural hematomas underwent MMA embolization using Optiblock coils. The baseline characteristics of the overall patient population are shown in Table 1. The mean patient age was approximately 74 years with a standard deviation of ~ 11 years, with predominant number of patients (83.9%) being male. The most common comorbidities included hypertension (HTN) (83.9%), and hyperlipidemia (HLD) (64.5%). Less frequent comorbid conditions included diabetes mellitus (DM) (19.4%), coronary artery disease (CAD) (25.8%), chronic kidney disease (CKD) (22.6%), atrial fibrillation (AFib) (25.8%), dementia (9/7%), and thrombocytopenia (3.2%). 21 patients (68%) presented with a new previously untreated SDH, 9 patients (29%) presented with recurrent SDHs, and one patient (3.2%) presented for prophylactic MMA embolization after SDH evacuation. 14 patients (45%) were on antiplatelets and/or anticoagulants upon presentation, of those 6 patients (50%) were on anticoagulants, including direct oral anticoagulants (DOACs) and warfarin, and 4 patients (33%) were on dual antiplatelet therapy. A total of 4 (13%) patients were on aspirin-only therapy.

**Table 1:** Patient demographics and comorbidities

Characteristics	Values
*N*	31
Age (Mean (SD))	72.32 (10.51)
Male Gender (n, %)	26 (83.9)
Hypertension (n, %)	26 (83.9)
Hyperlipidemia (n, %)	20 (64.5)
Diabetes Mellitus (n, %)	6 (19.4)
AFib/AFlutter (n, %)	8 (25.8)
Thyroid Disease	5 (16.1)
Coronary Artery Disease (n, %)	8 (25.8)
Cancer (n, %)	5 (16.1)
Chronic Kidney Disease (n, %)	7 (22.6)
Dementia (n, %)	3 (9.7)
Thrombocytopenia (n, %)	1 (3.2)
HIV/HCV (n, %)	1 (3.2)
DVT (n, %)	1 (3.2)
Antiplatelet/Anticoagulant Use (n, %)	14 (45)
Anticoagulant Use (n, %)	6 (19)
Aspirin Use only(n, %)	4 (13)
Dual Antiplatelet Therapy (n, %)	4 (13)

Table 2 describes the summary of characteristics of the SDHs and intraoperative radiation exposure. Most patients presented with unilateral hematomas (74%) with 65% of those unilateral hematomas being located in the left hemisphere. Only 8 patients (33%) presented with bilateral hematomas, all of whom underwent bilateral MMA embolization. Hematomas were most commonly located in the frontal (39.3%) or frontoparietal (32.2%) regions. The mean fluoroscopy time was 17.5 min (SD 9), and the mean radiation skin dose was 50,576 mGy (SD 45000) (Table 2). There were no intraoperative and immediate postoperative complications noted after the procedure. Additionally, there were no instances of delayed procedural complications or neurologic deterioration in this study. Only 2 patients (6.5%) required repeat surgical intervention after initial isolated MMA embolization to symptomatic enlargement of SDH. Two deaths (6.5%) were observed in this study over the follow-up period; however, all were a result of complications of the patients’ known co-morbid cardiovascular disease and unrelated to the embolization procedure. No deaths were reported that were associated with peri-procedural complications or SDH expansion.

**Table 2:** Summary of SDH characteristics and radiation exposure

Characteristic		Values
*N*		31
Embolization Laterality (n,%)	Left	15 (47)
	Right	8 (27)
	Bilateral	8 (33)
Hematoma Location	Frontal	11 (39)
	Frontoparietal	10 (32)
	Parietal	5 (18)
	Temporal	2 ( 7.1)
Procedure time (min) (mean, SD)		71.8 (50)
Fluoroscopy Time (min) (mean, SD)		17.49 (9)
Radiation Skin Dose (mGy) (mean, SD)		50,576 (44791)

A total of 39 embolizations were performed, the parameters of which are summarized in Table 3. On average, 1.28 ± 0.46 Optiblock coils were deployed per case, with a mean total coil length of 27.2 ± 16.4 cm per embolization. In the majority of procedures (72%), a single Optiblock coil was used. Two Optiblock coils were required in 28% of embolizations. The average length of each Optiblock coil used was 18.2 ± 7.9 cm. The most frequently used coil size was 3.5 mm × 20 cm (49%), followed by 3.5 mm × 30 cm (23%) and 3 mm × 10 cm (15%). Adjunctive embolization materials were used in all cases, most commonly embospheres (87%), with less frequent use of nBCA (10%) and non-Optiblock coils (2.6%). For the majority of cases (87%), the Optiblock coil was first deployed in a branch of the MMA, most commonly the frontal branch (65%) and less often the parietal branch (35%). Exclusive deployment in the common trunk of the MMA occurred in only 13% of cases. Intraoperative imaging demonstrated final vessel occlusion with coil placement most often involving the common trunk with one side branch (62%), followed by coil placement in common trunk and both branches (26%). On post-embolization angiographic runs of the external carotid artery, no contrast filling of middle meningeal artery was seen past the coil mass in any of the cases.

**Table 3:** Summary of intraoperative parameters during MMA embolization procedure

Number of Embolizations	39
Number of Coils (mean, SD)	1.28 (0.46)
Total Optiblock Coil Length Per Embolization (cm) (mean, SD)	27.24(16.42)
Number of Optiblock Coils Per Embolization	
One Coil (n(%))	28 (72)
Two Coil (n(%))	11 (28)
Total Optiblock Coil Length Per Embolization (cm) (mean, SD)	27.24 (16.42)
Average Length of a Single Optiblock Coil (cm) (mean, SD)	18.2 (7.9)
Specific Optiblock Coil Lengths Used	
3.5 mm x 20 cm (n(%))	19 (49)
3.5 mm x 30 cm (n(%))	9 (23)
3 mm x 10 cm (n(%))	6 (15)
Adjunct Embolization Materials	
Embospheres (n(%))	34 (87)
nBCA (n(%))	4 (10)
Non-Optiblock Coils (n(%))	1 (2.6)
Vessel in which coil was first deployed	
Frontal branch of MMA (n(%))	22 (56)
Parietal branch of MMA (n(%))	12 (31)
Common trunk of MMA (n(%))	5 (13)
Vessels occluded with Optiblock Coils	
Common trunk, and both branches of the MMA (n(%))	10 (26)
Common trunk of MMA and one side branch (n(%))	24 (62)
Common trunk of MMA only (n(%))	5 (13)

At presentation, the most common symptoms were headache (45.2%), gait instability (40.0%), weakness (35.5%), and AMS (29%) (Table 4). By the first follow-up, the prevalence of nearly all symptoms had declined markedly. Headache decreased from 45.2% to 13% (*p* < 0.01), weakness from 35.5% to 8.7%, and aphasia from 22.6% to 0%. Gait instability and AMS, present in 35.5% and 29% at baseline, were observed in only one and two patients, respectively, at first follow-up, and had completely resolved by the final visit. At last follow-up, only one of 12 patients with available data reported persistent symptoms, specifically headaches. Overall, these findings highlight substantial and durable symptomatic improvement following intervention, with statistically significant reductions in headache as early as the first follow-up which persisted to last follow-up.

**Table 4:** Comparison of presenting symptoms, symptoms at first follow-up, and symptoms at last follow-up“*‘–’ indicates that mcnemar’s test could not be performed because the number of discordant pairs was too small for statistical analysis to be valid*”

Symptom	Presenting	First_Fu_Sx	Last_Fu_Sx	McNemar’s Test *p*(Presenting, First_Fu_Sx)	McNemar’s Test *p*(Presenting, Last_Fu_Sx, )
Headache	14 (45.2)	3 (13.0)	1 (8.3)	< 0.01	< 0.05
Gait Instability	11 (35.5)	1 (4.3)	0 (0.0)	–	–
AMS	9 (29.0)	2 (8.7)	0 (0.0)	–	–
Dizziness	1 (3.2)	1 (4.3)	0 (0.0)	–	–
Seizure	1 (3.2)	0 (0.0)	0 (0.0)	–	–
Weakness	11 (35.5)	2 (8.7)	0 (0.0)	0.08	–
Aphasia	7 (22.6)	0 (0.0)	0 (0.0)	–	–

**Fig. 3:**
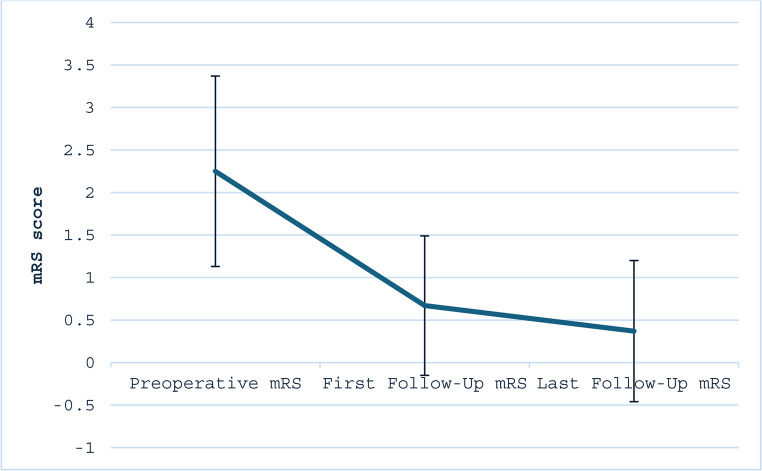
Comparison of mRS at presentation, first follow-up, last follow-up

Functional outcomes, as measured by the mRS, demonstrated significant improvement following intervention. First-follow up was on average 36 days post-operatively, while last follow-up was on average 91 days post-operatively. The mean preoperative mRS was 2.17 ± 1.23, which decreased to 0.55 ± 0.74 at first follow-up and further improved to 0.08 ± 0.29 at last follow-up (Table 5; Fig. 3). Comparison across time points using the Friedman rank-sum test confirmed a statistically significant reduction in disability scores (*p* < 0.001, *N* = 19). These findings indicate sustained functional recovery in the majority of patients over the follow-up period. No patients in the study had a decline in their mRS scores over the study period.

**Table 5:** Comparison of mRS at presentation, first follow-up, last follow-up

Characteristic	Overall
N	31
Preoperative mRS	2.17 (1.23)
First Follow-Up mRS	0.55 (0.74)
Last Follow-Up mRS	0.08 (0.29)
Friedman Rank-Sum Test	< 0.001*
	*N = 12

**Fig. 4:**
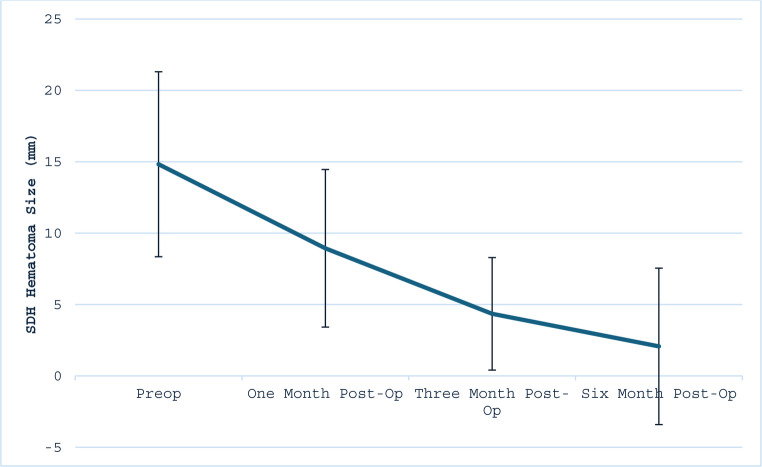
Mean subdural hematoma size on CT scan pre-operatively and at 1-, 3-, and 6- month follow-up intervals

**Fig. 5:**
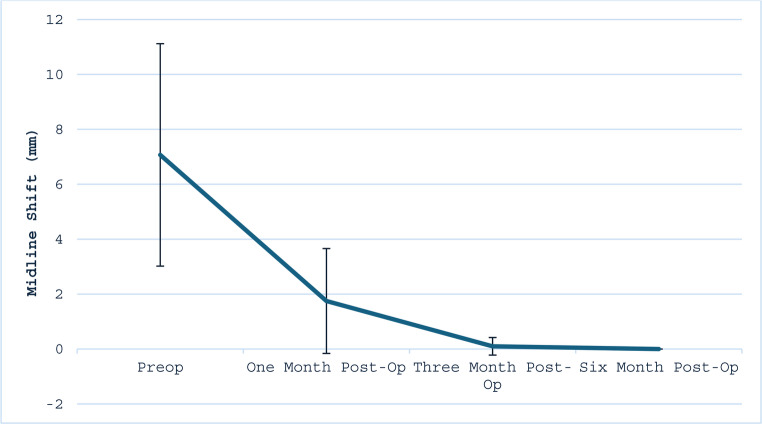
Midline Shift on CT scan pre-operatively and at 1-, 3-, and 6- Month Follow-Up Intervals

Serial imaging demonstrated a progressive reduction in hematoma size and midline shift. Mean subdural hematoma thickness decreased from 14.83 mm (SD 6.48) preoperatively to 8.94 mm, 4.35 mm, and 2.07 mm at 1-, 3-, and 6-month follow-up, respectively (*p* < 0.05) (Table 6; Fig. 4). This corresponds to a 40%, 71%, and 86% reduction in subdural hematoma thickness at one-, three-, and six-month follow-up, respectively, when compared to baseline measurements. Midline shift similarly declined from a preoperative mean of 7.07 mm (SD 4.05) to 1.75 mm, 0.10 mm, and 0.00 mm at the same respective intervals (*p* = 0.12) (Table 6; Fig. 5). Only two patients demonstrated slight radiographic enlargement of hematoma during follow-up. Both patients had bilateral subdural hematomas. In one case, while the left sided SDH completely resolved, the right-sided SDH size increased slightly from 15 mm to 17 mm at six months. Similarly, the second patient had a slight increase in the size of their right SDH from 16 mm to 17 mm one month after embolization. Neither required surgical reintervention, and both remained clinically stable.

**Table 6:** Mean subdural hematoma size and midline shift at 1-, 3-, and 6- month follow-up intervals

Timepoint	SDH Size (mm)	*p*-value compared to pre-op	SDH MidlineShift (mm)	*p*-value compared to pre-op	*n*
Preop	14.83 (6.48)	-	7.07 (4.05)	-	31
One Month Post-op	8.94 (5.52)	0.004	1.75 (1.91)	0.005	27
Three Month Post-op	4.35 (3.94)	*p* < 0.001	0.10 (0.32)	*p* < 0.001	15
Six Month Post-op	2.07 (5.48)	*p* < 0.001	0.00 (0.00)	*p* < 0.001	7
Friedman Rank-Sum Test	< 0.05		0.12		

## Discussion

This retrospective study evaluates the safety and efficacy of MMA embolization using Optiblock coils in the management of cSDH. Our findings demonstrate that coil-based embolization, augmented in most cases with embospheres, results in significant clinical, radiographic, and functional improvement with a favorable safety profile and a low rate of surgical reintervention.

The patient cohort was representative of the typical cSDH population, with a mean age of 74 years and a high prevalence of hypertension and hyperlipidemia—both known risk factors for vascular fragility and recurrence of subdural collections. No intraoperative, immediate or delayed complications were reported, and no procedure-related or SDH-related deaths were recorded.

This study presents the first dedicated clinical series evaluating the use of Optiblock coils for MMA embolization in the treatment of chronic subdural hematomas. Our findings demonstrate that embolization using Optiblock is associated with high rates of clinical improvement, functional recovery, and radiographic resolution, with minimal complications and a low rate of recurrence.

Symptom resolution was both rapid and durable. Most patients experienced significant improvement in presenting complaints, headache, gait instability, and AMS, as early as the first follow-up visit one month post-operatively. These improvements were mirrored by sustained gains in functional independence, as quantified by mRS scores, which decreased significantly over time. Importantly, no patients demonstrated clinical deterioration or mRS worsening post-embolization, underscoring the safety of these coils.

Serial CT imaging revealed continuous reductions in hematoma thickness and midline shift over the follow-up period, consistent with prior studies using particle- and liquid-based embolic agents. 94% of our patients demonstrated significant radiologic improvement in the size of their SDH after embolization, with approximately an 86% decrease in SDH size at 6 months post-operatively. Only two patients experienced marginal hematoma growth on follow-up imaging, and neither experienced clinical worsening or required further treatment. Only two patients (6.5%) required interval surgical drainage after initial isolated MMA embolizations, which aligns with historical rates associated with particle and liquid embolics alone, which range from 5 to 10% in some series [22–25]. Overall, these data suggest that use of Optiblock coils for MMA embolization in SDH is likely a safe and effective alternative or adjunct to particle and liquid embolics. Other reports in the literature have also supported this claim that coils, alone and as an adjunct to other embolics, are a safe and effective method for MMA embolization [18, 19, 26–29]. While particle and liquid agents remain widely reported for MMA embolization, their use can be limited by distal migration and risks such as cranial nerve injury [30]. Coils offer a predictable, purely mechanical mechanism of occlusion with excellent fluoroscopic visibility. Coils are particularly useful in cases with challenging anastomotic anatomy for example, proximity to the ophthalmic artery, where the risk of visual complications is heightened, often requiring abandonment of the procedure.

The Optiblock coil is a novel embolic platform optimized for mechanical occlusion of small-caliber vessels such as the MMA. Its integrated anchor–basket–fill architecture provides immediate proximal stability, broad luminal coverage, and a thrombogenic filler component, enabling high packing density. Extended coil lengths, up to 65 cm, further reduce the number of deployments required for durable vessel closure. In our cohort, a single Optiblock coil sufficed in most procedures (72%), with an average deployed length of 27 cm per embolization; the most frequently used sizes were 3.5 mm × 20 cm and 3.5 mm × 30 cm, reflecting the utility of longer coils in engaging the frontal and/or parietal branches as well as the common trunk.

Furthermore, the safety, simplicity and accuracy associated with coil takedown can increase its adoption under local or conscious sedation, obviating the need for general anesthesia and associated risks. Several other “solid” coil systems have been developed for vessel occlusion. The Penumbra POD and Amplatzer Vascular Plug perform well in medium- to large-vessel takedown but require larger 0.025-inch delivery systems, limiting use in smaller or tortuous distal arteries [31–33]. More recently, long-segment coils such as SwiftPAC have emerged as an alternative that can be delivered through 0.0165-inch microcatheters designed for improved packing density [34]. Early reports in the literature, including a study by Webb et al. which reported a mean deployed coil length of 15 cm and an average of 2.5 SwiftPAC coils per case, highlight a growing interest in long, mechanically efficient coils for MMA embolization [26]. In our current experience, Optiblock coils are an even more efficient solution with a single coil used in most cases, with the advantages of engineered coil components with the goal of vessel occlusion. Since this experience with Optiblock coils, there are now options for sizes such as 2 × 20 cm and 2.5 × 20 cm suited for smaller MMAs. All the coils in our series were deployed through an 0.0165 SL-10 (Stryker) microcatheter with no difficulty in deployment. From a cost perspective, a single Optiblock coil is generally comparable in price to the ~ 0.5 cc of Onyx often used for MMA embolization, yet is likely substantially more economical than coil-predominant strategies requiring multiple shorter conventional coils. The ability to achieve durable vessel occlusion with a single long, integrated coil therefore offers meaningful procedural, economic, and technical advantages.

Adjunctive particulate embolization is commonly employed to promote distal penetration, and since particles are not radiopaque, the coil also serves as a reliable fluoroscopic marker of the embolization site. In our series, final angiography most often demonstrated coil occlusion of the common trunk with one side branch (62%), with coil occlusion of the common trunk and both branches in 26% of embolization. The proximal coil occlusion of the middle meningeal artery in these cases was noted to be robust with no contrast opacification of MMA on post embolization angiographic injections of external carotid artery. These patterns are consistent with a workflow in which coils establish a controlled proximal occlusive mechanical scaffold and particles are used to complete unilateral distal branch occlusion, particularly when only one branch of the MMA is embolized with particles or liquid embolics. It is hypothesized that distal MMA penetration is critical, as the pathologic neovasculature driving persistent exudation and hematoma growth is supplied by small distal meningeal branches that feed the subdural membranes [35]. For this reason, the majority of cases in our series combined proximal coil occlusion with adjunctive embolic agents, most commonly embospheres, to achieve durable distal branch shutdown and reduce the risk of cSDH recurrence. Notably, the only case in which neither particles nor nBCA were used involved the presence of dangerous ophthalmic artery anastomoses, in which distal embolization was intentionally avoided to mitigate the risk of visual complications.

This study has several limitations. First, its retrospective, single-arm design without a control cohort limits the ability to determine whether Optiblock-based embolization offers comparative advantages over other embolic agents or surgical management. Second, the overall sample size is relatively small (*n* = 31), which reduces statistical power and increases the likelihood that observed treatment effects may be influenced by sampling variability. Third, follow-up attrition, particularly at later time points with only seven patients undergoing 6-month imaging, limits the reliability of long-term radiographic conclusions and may bias outcome estimates toward those who returned for follow-up. In addition, this is a heterogeneous dataset in which Optiblock coils were rarely used in isolation, making it difficult to generalize their standalone efficacy; only one patient in this cohort underwent coil-only embolization, underscoring the need for larger studies to more fully evaluate isolated proximal coil occlusion. A notable finding was the rapid and robust resolution of symptoms and improvement in functional status as measured by mRS scores, which appeared more pronounced than results reported in recent randomized trials using liquid embolics [36]. While this may partly reflect the small cohort size, it is also plausible that the durable proximal mechanical occlusion achieved by Optiblock coils, often combined with adjunctive particles, contributed to more sustained shutdown of the MMA. The coil’s long, integrated anchor–basket–fill design creates a dense, stable construct that may offer greater resistance to recanalization than shorter traditional coils or liquid-only techniques. However, given the retrospective nature of this study and the limited number of coil-only cases, these observations should be interpreted cautiously. Larger prospective comparative studies are needed to determine whether the favorable outcomes observed here reflect a true advantage in the durability of Optiblock-based occlusion or are attributable to sample size and patient selection.

Future prospective, comparative studies are warranted to evaluate the efficacy of Optiblock coils alone against other embolic agents, including particles and Onyx, and to better characterize the optimal patient selection and procedural strategy. Additionally, cost-effectiveness analyses could help determine the economic viability of Optiblock-based embolization in broader clinical practice.

## Conclusion

MMA embolization using Optiblock coils is a safe, efficient, and clinically effective treatment for patients with subdural hematomas. Our findings support its use as a promising alternative to traditional embolic platforms, particularly in settings where there is high risk of complications from use of liquid or particle embolics. This study supports use of these coils in a one-and-done fashion given small coil diameters aligned with long lengths, volumetric 14 primary wire construction promoting thrombogenesis, and engineering geared toward vessel takedown. In comparison, traditional saccular aneurysm coils are shorter, less likely to stack, and hence inefficient, requiring multiple coils for every treatment. Further prospective trials are needed to validate these results and define the optimal role of Optiblock coils within the evolving treatment paradigm for chronic subdural hematoma.

## Supplementary Information

Below is the link to the electronic supplementary material.

Supplementary Material 1 Supplementary Figure 1: Intraoperative video depicting endovascular deployment of a single 3.5 mm × 20 cm Optiblock coil, initiated in the parietal branch and resulting in occlusion of both branches and the common trunk.

## Data Availability

No datasets were generated or analysed during the current study.
